# Identifying the unique characteristics of the Chinese indigenous pig breeds in the Yangtze River Delta region for precise conservation

**DOI:** 10.1186/s12864-021-07476-7

**Published:** 2021-03-02

**Authors:** Qing-bo Zhao, Favour Oluwapelumi Oyelami, Qamar Raza Qadri, Hao Sun, Zhong Xu, Qi-shan Wang, Yu-chun Pan

**Affiliations:** 1 School of Agriculture and Biology, Department of Animal Science, Shanghai Jiao Tong University, Shanghai, 200240 P.R. China; 2 Department of Animal Breeding and Reproduction, College of Animal Science, Zhejiang University, Hangzhou, 310030 P.R. China

**Keywords:** Chinese local pig breeds, Unique characteristics, Precise conservation, Partial least squares, Modules

## Abstract

**Background:**

China is the country with the most abundant swine genetic resources in the world. Through thousands of years of domestication and natural selection, most of pigs in China have developed unique genetic characteristics. Finding the unique genetic characteristics and modules of each breed is an essential part of their precise conservation.

**Results:**

In this study, we used the partial least squares method to identify the significant specific SNPs of 19 local Chinese pig breeds and 5 Western pig breeds. A total of 37,514 significant specific SNPs (*p* < 0.01) were obtained from these breeds, and the Chinese local pig breed with the most significant SNPs was Hongdenglong (HD), followed by Jiaxing black (JX), Huaibei (HB), Bihu (BH), small Meishan (SMS), Shengxian Hua (SH), Jiangquhai (JQ), Mi (MI), Chunan (CA), Chalu (CL), Jinhualiangtouwu (JHL), Fengjing (FJ), middle Meishan (MMS), Shanzhu (SZ), Pudong white (PD), Dongchuan (DC), Erhualian (EH), Shawutou (SW) and Lanxi Hua (LX) pig.

Furthermore, we identified the breeds with the most significant genes, GO terms, pathways, and networks using KOBAS and IPA and then ranked them separately. The results showed that the breeds with the highest number of interaction networks were Hongdenglong (12) and Huaibei (12) pigs. In contrast, the breeds with the lowest interaction networks were Shawutou (4) and Lanxi Hua pigs (3), indicating that Hongdenglong and Huaibei pigs might have the most significant genetic modules in their genome, whereas Shawutou and Lanxi Hua pigs may have the least unique characteristics. To some degree, the identified specific pathways and networks are related to the number of genes and SNPs linked to the specific breeds, but they do not appear to be the same. Most importantly, more significant modules were found to be related to the development and function of the digestive system, regulation of diseases, and metabolism of amino acids in the local Chinese pig breeds, whereas more significant modules were found to be related to the growth rate in the Western pig breeds.

**Conclusion:**

Our results show that each breed has some relatively unique structural modules and functional characteristics. These modules allow us to better understand the genetic differences among local Chinese and Western pig breeds and therefore implement precise conservation methods. This study could provide a basis for formulating more effective strategies for managing and protecting these genetic resources in the future.

**Supplementary Information:**

The online version contains supplementary material available at 10.1186/s12864-021-07476-7.

## Background

There is a large number of indigenous pig breeds in China. Effective protection of these breeds is related to the pig industry’s sustainable development and is of great significance to protect genetic diversity globally. Through thousands of years of artificial domestication and natural selection, most of these pigs have developed various genetic characteristics.

For example, the Taihu pig was a single breed before 1974, but it is now divided into seven breeds based on unique features or characteristics. These seven pig breeds are Jiaxing black, Erhualian, Fengjing, Shawutou, Meishan, Mi, and Hengjing, which is now extinct. These pig breeds are all world-famous for their high reproductive capacity. Some other local pig breeds have excellent meat quality, such as Jinhua pigs and Dongchuan pigs. The meat of these two pig breeds is very suitable for ham production in China. Additionally, we investigated other pig breeds with special characteristics in this study. Bihu pigs, Lanxi Hua pigs, and Shengxian Hua pigs are highly adaptable and resistant to rough feeding. Chunan is also famous for its meat because of its fresh colour, juiciness, fragrant taste, and tender quality. It is also a high-quality raw material for cured ham and bacon. The coat colour of some pig breeds, such as Jiaxing black, Chalu black, Bihu, Meishan, and Dongchuan, is black, whereas Pudong white pigs are white, and the coat colour of most Shengxian Hua pigs is between greyish-brown and white [1]. In short, we know that most local pig breeds have excellent characteristics, such as high fecundity, strong adaptability, and good meat quality, but their unique characteristics should also be investigated.

There are approximately 108 local pig breeds [1] and strains in China, and the effective protection of all of these breeds would contribute to the sustainable development of China’s pig industry and the richness of the world’s domestic animal resources. In particular, research on each breed’s unique structural modules and characters will aid in the formulation of protection plans for each breed based on their local conditions and facilitate their specific conservation. Preserving the unique variations, genes, modules, and characteristics of each breed is extremely important for maintaining biodiversity and adapting to future environmental changes.

Various characteristics of domestic animals in long-term natural and artificial selection will leave corresponding genetic imprints on their genomes. These genetic imprints are often referred to as selection signals. The study of selection signals is a research strategy based on the genome-to-phenotype concept. Given the lack of phenotypic records and the small population size of indigenous pig breeds in China, it has become an increasingly important method for analysing livestock germplasm characteristics. For example, using resequencing data, Li et al. [2] (2003) performed a genome-wide scan to detect genes related to hypoxic adaptability, olfaction, energy metabolism, and drug response in Tibetan pigs, revealing various genes of economic importance that might be subject to long-term selection. These genomic imprints also revealed the genetic adaptation of Tibetan pigs to high altitudes. Wang et al. [3] (2015) performed a whole-genome selection signal detection analysis and revealed genes related to fur colour and reproductive traits in Chinese Tongcheng pigs. Furthermore, Ai et al. [4] (2015) conducted a genome-wide scan of 69 pig breeds from 15 different geographical locations in China and discovered a set of loci that may be responsible for their adaptation to high and low altitudes, providing a basis for studying the evolutionary history and gene introgression of pigs. Zhao et al. [5] (2018) also revealed evidence of evolutionary changes in the genetic and phenotypic characteristics of Meishan pigs using a selective sweep strategy. However, all the studies mentioned above were designed to investigate whether genetic variations or signatures of selection exist among local Chinese pig breeds and paid less attention to unique characteristics. Therefore, there is a need to identify each breed’s unique characteristics, as this would help design strategies to manage and conserve these genetic resources effectively. This research is particularly useful when designing specific conservation programs for each indigenous pig breed.

There are also several methods for identifying differences among populations. Chen et al. [6] (2016) proposed a new method called EigenGWAS to find loci under selection using the eigenvectors in a structured population. This method was also utilized by Zhao et al. [7] (2018) to identify the differences between two chicken breeds. Sun et al. [8] (2019) proposed a novel method that utilizes partial least squares (PLS) to identify differences among populations. This method’s main advantage is that the principal component and response variable must maintain the maximum correlation in extracting the principal component. The PLS method has indicated to be an efficient statistical regression technique because it combines both principal component analysis (PCA) and correlation analysis [8]. Moreover, under some conditions, the PLS method has been proven to have better effects than Fst [9], which is also a prevalent method for identifying population differentiation.

Therefore, in this study, we used the PLS method to analyse five Western pig breeds and nineteen local Chinese pig breeds in the Yangtze River Delta region of China to explore their relatively unique characteristics that formed due to long-term selection, laying the foundation for their precise future conservation.

## Results

### SNP distribution

**Fig. 1:**
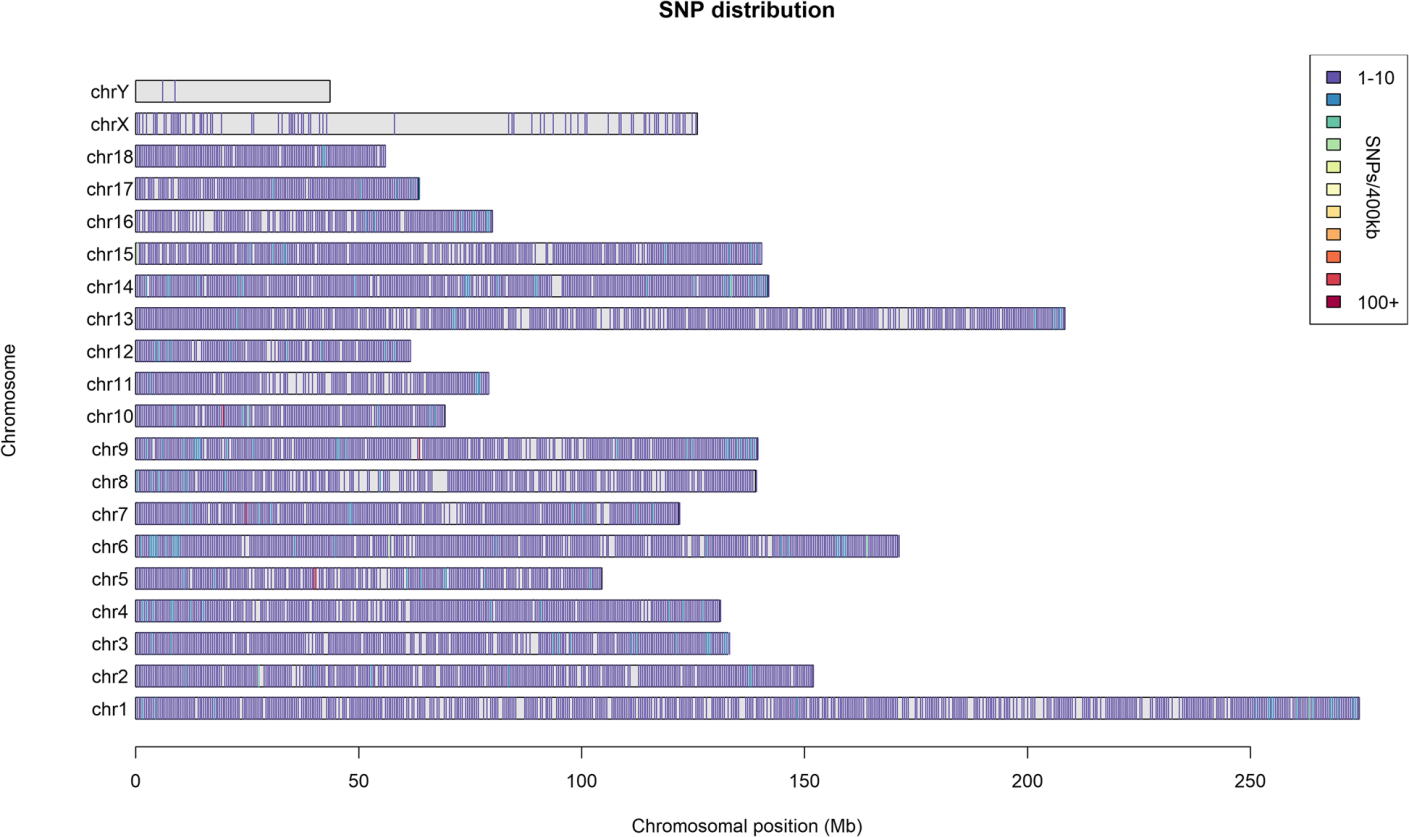
The distribution of SNPs on each chromosome. The horizontal axis shows chromosome length (Mb); the different colors represent SNP density per 400 kb window size

We analysed the distribution of SNPs on each chromosome using a 400-kb non-overlapping window size. The results showed that the SNPs were evenly distributed across the entire genome except for the sex chromosomes (Fig. 1).

### PCA, PCoA, and t-SNE

**Fig. 2:**
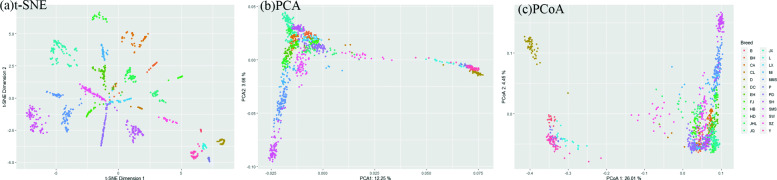
t-SNE, PCA and PCoA plot for all breeds

First, we used t-SNE to best classify the populations to perform dimensionality reduction clustering analysis on all the breeds. From Fig. 2a, we can see that each breed is well clustered. Furthermore, we used the PCA and PCoA methods to extract the first and second principal components, respectively, and plotted them. Among these two methods, the PCA’s first principal component can explain 12.25% of the total variation and the second principal component can explain 3.66% of the total variation (Fig. 2b). In comparison, for the PCoA method, the corresponding first and second principal components can explain 26.01 and 4.45% of the total variation (Fig. 2c), respectively.

We can distinguish the five Western pig breeds from the 19 Chinese pig breeds through the first principal component. Compared with the other four Western breeds, Yorkshire pigs (Y) are more dispersed and closer to the local Chinese breeds (Fig. 2b). Through other principal components, other breeds can also be distinguished in sequence. Overall, these breeds are relatively independent units of genetic resources.

### Significant specific SNPs of each breed

**Fig. 3:**
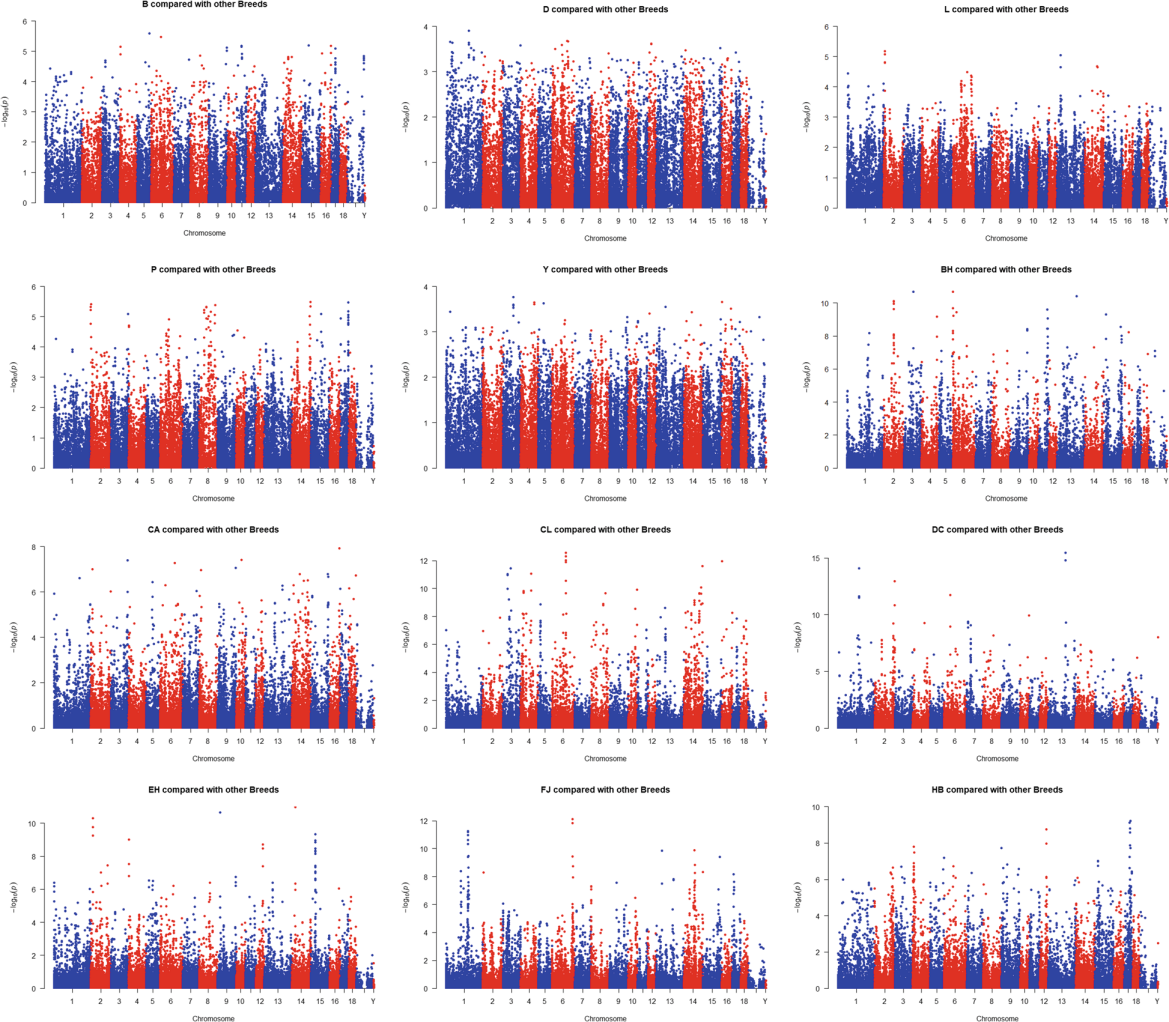
The Manhattan plots for each of 12 breeds (B D L Y P BH CA CL DC EH FJ HB) compared to the other breeds

**Fig. 4:**
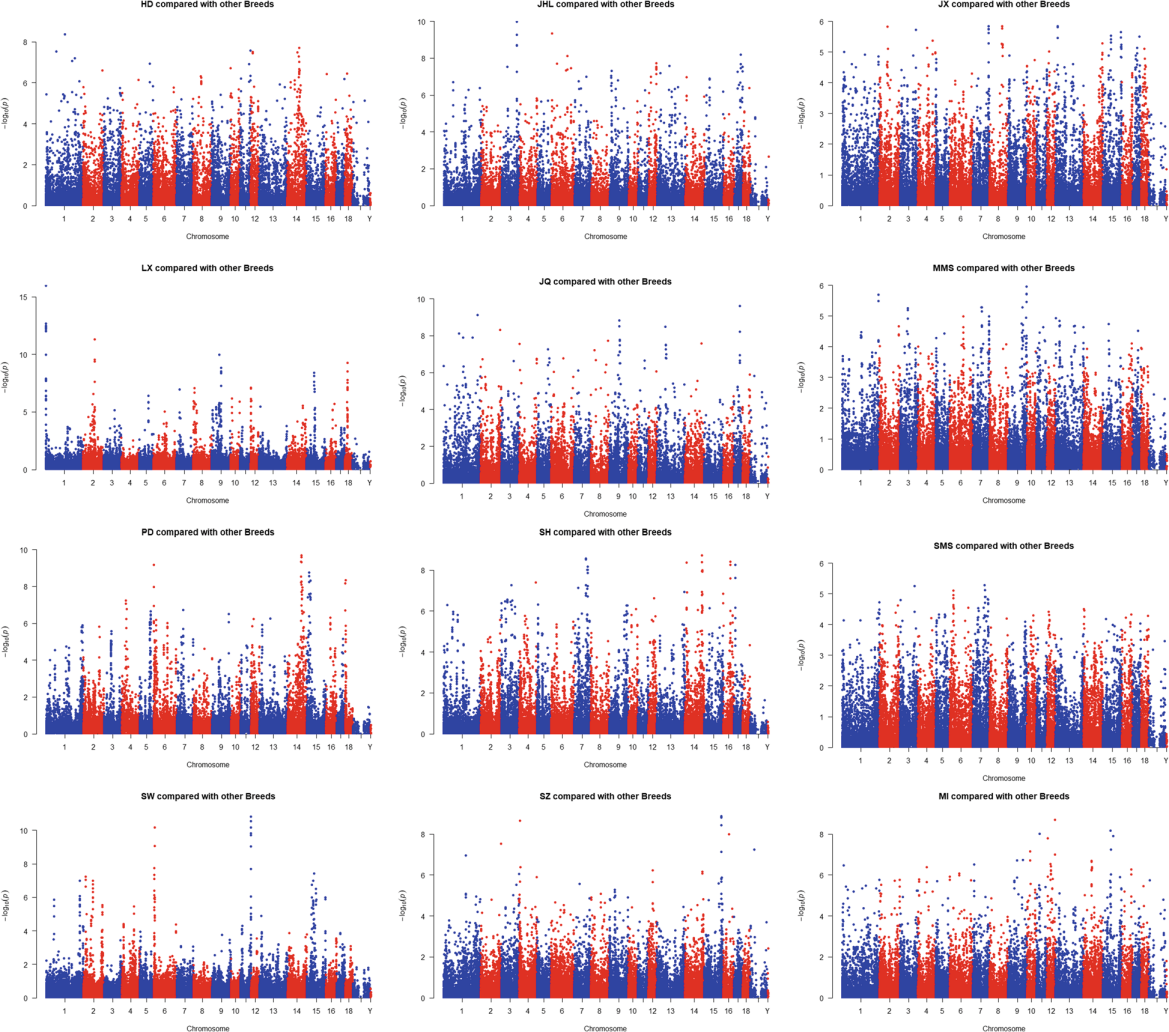
The Manhattan plots for each of 12 breeds (HD JHL JX LX JQ MMS PD SH SMS SW SZ MI) compared to the other breeds

Among these 24 breeds, we found a total of 37,514 significant SNPs (*P* < 0.01). The number of significant SNPs corresponding to each breed is shown in Table 2. The breeds with the most significant SNPs were Hongdenglong (HD), followed by Jiaxing black (JX), Huaibei (HB), Bihu (BH), small Meishan (SMS), Shengxian Hua (SH), Jiangquhai (JQ), Mi (MI), Chunan (CA), Chalu (CL), Jinhualiangtouwu (JHL), Fengjing (FJ), middle Meishan (MMS), Shanzhu (SZ), Pudong white (PD), Dongchuan (DC), Erhualian (EH), Shawutou (SW) and Lanxi Hua (LX). Manhattan plots of the -log (*p*) value corresponding to each locus for each breed after PLS analysis are shown in Figs. 3 and 4.

### Functional annotation and enrichment analysis

First, we mapped the significant SNPs obtained from the 24 breeds to the corresponding genes. In general, the number of genes corresponding to each breed’s significant SNPs was roughly the same as the number of significant SNPs found in each breed, but there were also subtle differences. The breeds with the highest number of significant genes in descending order are Huaibei, Hongdenglong, Jiangquhai, Bihu, small Meishan, Berkshire, Jiaxing black, Fengjing, Pietrain, Chunan, Duroc, Mi, Shengxian Hua, Shanzhu, Pudong white, middle Meishan, Landrace, Dongchuan, Yorkshire, Jinhualiangtouwu, Chalu, Erhualian, Shawutou, and Lanxi Hua pigs. We can observe that the number of significant genes mapped is relevant to each breed’s significant genes but is not the same. All the specific significant genes of each breed are shown in Additional file 1. In particular, we found that the most significant genes in the Bihu pig breed, *ALPK2, SHROOM4, GRID1, GLI2,* and *ERCC3*, are related to heart morphogenesis, brain development, social behaviour, cardiac development, lung development, and hair cell differentiation. The most significant genes in the Chuanan pig breed, *FAR2, FA2H, PTPRJ, PRXL2A,* and *ATP8A2*, are related to lipid metabolic processes, fatty acids, negative regulation of vascular permeability, antioxidant activity, and ageing, respectively. Furthermore, the most significant genes in the Dongchuan pig breed, *SMAD6, MAP2K5, NXNL1,* and *PTPRJ*, are associated with the immune response, phosphorylation and heart development, cell redox homeostasis, and cell growth regulation, respectively. In the Erhualian pig breed, we found that the most significant genes *ALPK2, PTPN3,* and *PALM2AKAP2* are related to the regulation of apoptotic cells, the cell cycle, and cell shape, respectively. The *SMAD3* gene found in the Erhualian pig breed is related to multiple functions, such as cell growth regulation, liver development, the hypoxia response, and the immune response. In the Huibei pig breed, the most significant genes, *SMAD6, VCAN, CD44, EXT1,* and *GNA12*, are associated with the immune response, central nervous system development, cartilage development, olfactory bulb development, and cell differentiation, respectively. In the Hongdenglong pig breed, we found that the most significant genes, *MEDAG, IMMP2L, BMPR1A,* and *MAP2K1,* are related to fat cell differentiation, follicle development, the immune response, and the hypoxia response, respectively. In the Jinhualiangtouwu pig breed, we found that the most significant genes, *ITGA9, ITPR2,* and *DGKZ*, are associated with cell adhesion, the hypoxia response, and lipid phosphorylation, respectively. In contrast, the *GLI2* gene detected in this breed is associated with cell differentiation, lung development, and mammary gland development. The above results suggest that most of the significant genes found in each local Chinese pig breed are related to reproduction, meat quality, and strong adaptability.

Among the Western pig breeds, we found that the most significant genes *FOXK1, SERINC5,* and *ROBO2* are related to glucose metabolism and the starvation response, the innate immune response, and the hormone stimulus response in the Duroc breed, respectively. In the Landrace breed, we found that the most significant genes, *SASH1, RPL26L1, CARD11, HNF4A,* and *ERCC2*, are associated with the regulation of protein autoubiquitination, structural constituent of ribosome, immunoglobulin production, sex differentiation, and DNA repair, respectively. We found that the most significant genes in the Yorkshire breed, *LDLRAD4, ATP8A2, PLCD1, ST13,* and *MYD88*, are associated with cell migration, ageing, lipid metabolism, heat shock protein binding, and the inflammatory response, respectively. In the Pietrain breed, we found that the most significant genes, *CLOCK, SPATA18, SLC1A2, FSHR,* and *OAS2,* are related to inflammation, DNA damage stimulus, the drug response, ovarian follicle development, and the immune response, respectively. In general, the SNPs found in each breed of the local Chinese pig breeds were more significant than the SNPs found in the Western pig breeds. However, some of the most significant SNPs in the breeds could not be mapped to corresponding genes because few genes have been annotated in pigs. Therefore, further research should link these SNPs to specific genes and traits in Chinese pigs.

We further performed enrichment analysis on the significant genes obtained in the previous step to generate GO terms, KEGG pathways, and molecular networks. The largest numbers of GO terms (*p* < 0.05) were found in MMS, B, SMS, BH, SH, CA, FJ, JHL, Y, SZ, P, JQ, MI, CL, HD, HB, PD, D, SW, JX, L, DC, EH, and LX in decreasing order (Table 1 and Additional file 2). The number of GO terms with a value of *p* < 0.01 for each breed was similar to the number of GO terms with *p* < 0.05 except for a few breeds, such as the small Meishan and Bihu pig breeds. Similarly, if sorted according to the number of pathways (*p* < 0.05), the order was FJ, MMS, CL, JQ, HD, B, L, SH, JHL, Y, CA, HB, JX, DC, EH, BH, P, D, PD, MI, SW, SMS, SZ, and LX. The number of pathways (*p* < 0.01) in each breed was similar to the number of pathways with *p* < 0.05, and only in Hongdenglong, Shawutou, and a few other breeds was there a minor difference. Interestingly, the Shawutou pig breed has the characteristic of easily gaining weight and becoming fat. This characteristic is supported by a GO term (“GO:0071363”) and could be related to animal weight gain.

**Table 1:** Breed name, abbreviation, population size, and region of all breeds

Region	Breed	Code	Size
Western	Duroc	D	49
	Landrace	L	21
	Yorkshire	Y	53
	Pietrain	P	20
	Berkshire	B	16
Jiangsu	Small Meishan	SMS	75
	Mi	MI	36
	Erhualian	EH	42
	Dongchuan	DC	10
	Huaibei	HB	34
	Hongdenglong	HD	30
	Jiangquhai	JQ	38
	Shan	SZ	20
Zhejiang	Bihu	BH	30
	Chunan	CA	59
	Chalu	CL	22
	Jinhualiangtouwu	JHL	57
	Lanxi	LX	40
	Shengxianhua	SH	64
	Jiangxing Black	JX	91
Shanghai	Middel Meishan	MMS	97
	Shawutou	SW	65
	Fengjing	FJ	32
	Pudong White	PD	68

**Table 2:** The number of significant SNPs, Genes, GO Terms, KEGG Pathways, and Networks

Breed	Sig SNPs (***P*** < 0.01)	Sig Genes (***P*** < 0.01)	GO Term (***P*** < 0.05)	GO Term (***P*** < 0.01)	KEGG Pathway (***P*** < 0.05)	KEGG Pathway (***P*** < 0.01)	Networks
D	1686	100	16	4	7	2	8
L	1383	80	4	0	30	9	6
Y	1077	70	55	24	17	4	8
P	1670	102	41	9	7	0	10
B	2047	112	166	63	34	13	10
SMS	1835	113	96	39	3	1	11
MI	1756	96	31	14	5	1	8
EH	1168	61	0	0	14	1	5
DC	1221	75	2	0	15	5	6
HB	1997	144	22	3	15	6	12
HD	2062	143	26	2	43	22	12
JQ	1757	129	33	2	44	20	9
SZ	1405	85	52	27	3	1	9
BH	1947	120	92	22	10	2	10
CA	1720	101	77	15	16	5	9
CL	1661	66	27	4	53	21	6
JHL	1559	67	70	19	23	3	5
LX	645	35	0	0	1	0	3
SH	1822	95	80	22	26	5	7
JX	2038	111	6	0	15	5	8
MMS	1501	83	320	152	55	25	6
SW	650	40	9	1	4	3	4
FJ	1518	110	77	18	59	25	9
PD	1389	85	18	9	6	1	6

### Significant ingenuity pathway analysis of molecular networks

**Fig. 5:**
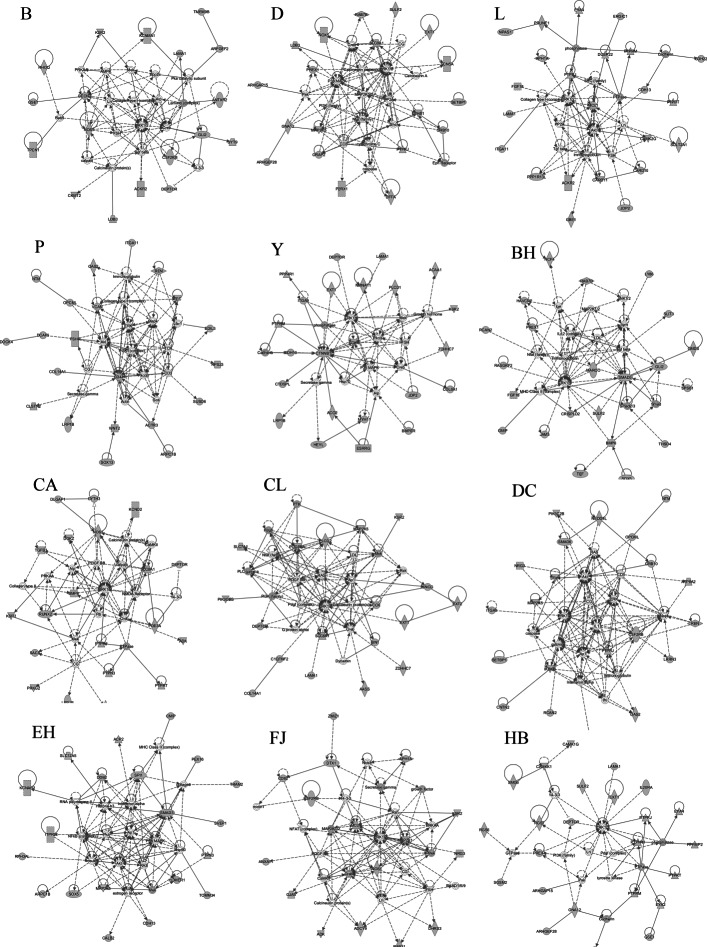
Significant ingenuity pathway analysis molecular networks for 12 breeds

**Fig. 6:**
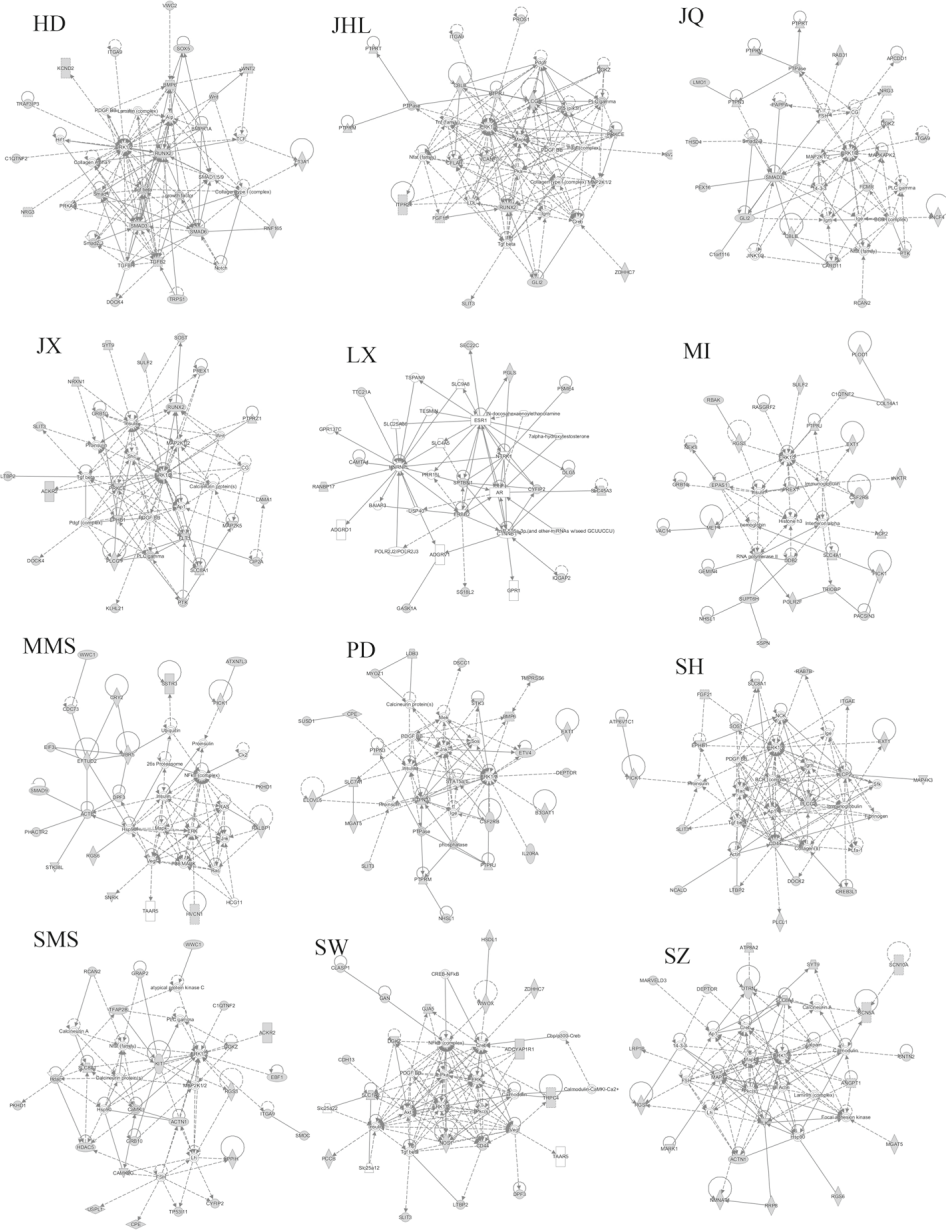
Significant ingenuity pathway analysis molecular networks for another 12 breeds

The breeds with the largest number of gene interaction networks were Hongdenglong and Huibei, with 12 networks, while the breed with the smallest number was Lanxi Hua, with only three networks. Graphs of the gene interaction networks with the ranking of each breed are shown in Figs. 5 and 6. More details about all molecular networks of each breed can be found in Additional file 3. The key molecular network of Bihu pigs in this study is related to cell development and function, connective tissue development and function, and bone and muscle system development and function. The Chunan Hua pig molecular network highlights important associations with cell morphology, organ damage and abnormalities, and bone and muscle system development. The most important molecular network features in the Chalu breed are related to cancer, connective tissue diseases, and developmental disorders. In Dongchuan pigs, the most important molecular network is cancer, intercellular signalling, and nervous system function. The most important molecular networks in Erhualian are related to cell assembly, connective tissue diseases, nervous system development and function. Furthermore, in the Fengjing breed, the most important molecular network is related to cardiovascular diseases, organ development, organ damage and abnormalities; in Huibei, the most important molecular network is related to cancer, gastrointestinal diseases, post-translational modification; the most important molecular network in Hongdenglong is related to cell development, connective tissue development and function, bone and muscle system development and function; the most important molecular network in Jinhualiangtouwu is related to cancer, gastrointestinal diseases, biological damage and abnormalities; the most important molecular network in Jiangquhai is related to the cell-mediated immunity response, cell development, cell function and maintenance; the most important molecular network in Jiaxing black is related to cardiovascular system development and function, cell movement, nervous system development and function; the most important molecular network in Lanxi Hua pigs is related to cell development, growth and proliferation, and tissue development; the most important molecular network in Mi pigs is related to cancer, gastrointestinal diseases, biological damage and abnormalities; the most important molecular network in middle Meishan pigs is related to cancer, connective tissue diseases and developmental disorders; the most important molecular network in Pudong white pigs is related to cell development, blood diseases, and tissue morphology; the most important molecular network in Shengxia hua pigs is related to cell morphology, cell movement, blood system development, and function; the most important molecular network in small Meishan pigs is related to cell function and maintenance, cell movement, and blood system development; the most important molecular network in Shawutou pigs is related to amino acid metabolism, molecular transport, and small molecule biochemistry; and the most important molecular network in Shanzhu pigs is related to cardiovascular diseases, nervous system diseases, organ damage, and abnormalities.

In the Western pig breeds, the most important molecular network in Berkshire pigs is related to cancer, cell and tissue assembly, biological damage and abnormalities; the essential molecular network in Duroc pigs is related to cancer, developmental disorders, biological damage and abnormalities; the most important molecular network in Landrace pigs is related to the development and function of the cardiovascular system, cell morphology and embryonic development; the most important molecular network in Pietrain pigs is related to carbohydrate metabolism, intercellular signalling and interaction, nervous system development and function; and the most important molecular network in Yorkshire pigs is related to the cell cycle, cell development and embryo development.

Specifically, we found some networks or structural modules related to digestive system development and function, regulation of cardiovascular and genetic diseases, and amino acid metabolism in local Chinese breeds. This finding indicates that local Chinese pig breeds may have better performance in rough feeding tolerance, disease resistance, and good meat quality than Western pig breeds. After long environmental adaptation and selection pressure, the local Chinese pig breeds might have gradually formed a corresponding genetic imprint or feature module on the genome for these characteristics. In the Western pig breeds, more pathways and networks (modules) related to cell growth were found, which may be related to the long-term selection pressure of the Western pig breeds related to the growth rate.

## Discussion

This study shows that all 24 populations can be characterized by using the t-SNE method, indicating differences among these pig breeds. Hence, our research used the partial least squares method to detect each breed’s characteristics. Simultaneously, when we performed principal component analysis, we found that the Western and Chinese local pig breeds were mainly partitioned by the first principal component and the second principal component. However, no separation was possible when the local pig breeds in China were studied. This finding is consistent with our subsequent results. These local Chinese pig breeds show high fertility, strong adaptability, and good meat quality as their distinctive features, whereas the Western pig breeds are mainly characterized as fast growth breeds.

We used the PLS method to detect the specific characteristics of each breed. It was confirmed that the PLS method more easily detects selection signals and has fewer false positives in a previous study [8]. Some studies have studied selection signals for one or several of these special local pig breeds. For example, Yang et al. [10] identified positive selection footprints mainly involved in the immune response and the development of tissues and organs by using the Fst method in six Chinese indigenous pig breeds, one developed breed and two commercial breeds. Li et al. [11] revealed several candidate genes associated with health, reproduction and meat quality in Jinhua pigs compared with nine other breeds by using the XP-EHH method. Diao et al. [12] detected the selection signals in three southern Chinese indigenous pigs and found some common genes using both XP-EHH and Fst methods. Different methods have different detection effects, and until now, there has been no research on the specific detection of each breed among many local pig breeds in China. In our study, we found some new genes and network modules related to immunity, reproduction, and meat quality by employing the PLS method as a supplement to other methods.

However, a disadvantage or limitation in this study is that we used Genotyping by Genome Reducing and Sequencing (GGRS) data, which is simplified genome data. This leads to the fact that the genome data we obtained is not as complete and same as the whole genome data, thus we may miss some important SNPs and specific clusters which could be related the main performance characteristics of some breeds. Also, there is a lack of comprehensive functional annotations of gene loci for Chinese local pigs till now, which further limited us to find more specific modules associated with important characteristics of the breeds in our study. The results we found are to assist our point of view of structure conservation. Through this research, we hope structural modules for conservation could be paid more attention in the future.

In short, in this study, we first identified significant SNPs and corresponding genes for each breed by using the partial least squares method. Then, each breed’s genes were enriched, and molecular networks of the interactions between genes were constructed to discover whether there were obvious structural or functional modules in each breed. We investigated the unique genetic characteristics of these breeds from a macroscopic perspective in this study. The genes, pathways, and networks of each breed may be relatively close, which is also why some of these breeds were combined as “one large breed” previously. However, with the genetic drift effect of small populations, each breed’s uniqueness will become increasingly apparent. Nevertheless, we still found some unique characteristics of these breeds, such as a GO term related to the fattening performance of Shawutou pigs. Genes, pathways, and networks related to immunity, reproduction, and meat quality were identified in most of the local Chinese pig breeds. However, we did not find any significant genes or pathways related to coat colour, possibly because we used simplified sequencing data and because different methods have different detection power.

Our results can provide a molecular basis for breeding managers and governments to conserve local pig breeds in China. Nevertheless, we only used existing methods, such as gene enrichment analysis, pathway analysis, and network analysis, to determine each breed’s unique genetic structure and functional unit. It is vital and essential to find better ways to distinguish each breed’s specific modules, including known functions or unknown functions, to realize precise protection and comprehensive protection of local pig breeds throughout China. The scope of this work can provide more insight into the conservation of breeds in future studies.

## Conclusion

Our results show that each breed does has some relatively unique structural modules and functional characteristics. Hongdenglong and Huibei pigs have the most significant genetic modules in their genome, whereas Shawutou and Lanxi Hua pigs have the least unique structural and functional characteristics. In general, more modules were found to be related to the development and function of the digestive system, regulation of diseases, and metabolism of amino acids in local Chinese pig breeds. However, most modules in the Western pig breeds were found to be related to the growth rate. These modules allow us to better understand the genetic differences between these breeds and implement precise conservation strategies. This study could provide a basis for formulating more effective strategies for the management and protection of these genetic resources.

## Methods

### Population and sequencing data

A total of 1069 pigs were included in this study, of which 159 were Western breeds, including five breeds: Duroc, Landrace, Yorkshire, Pietrain, and Berkshire. The remaining 910 pigs were local Chinese pig breeds from the Yangtze River Delta region. All the pigs were selected from different pig farms in China (see Additional file 4). More information on all the pig breeds, including the breed name, abbreviation, population size, and region of origin, can be found in Table 1. The sequencing data of most of the individuals in this study were from previous studies [11, 13–15], and the sequencing data of other individuals were obtained by genotyping with a genome reducing and sequencing (GGRS) strategy [16] using these pig ear tissue samples. SAMtools software [17] (version 0.1.19) was used to call SNPs, after which the missing genotype data were imputed by using Beagle (version 5.0) software [18]. We finally obtained a total of 62,822 SNPs with a minor allele frequency (MAF) ≥ 0.05 for subsequent analysis.

### Principal component analysis, principal coordinates analysis and t-distributed stochastic neighbour embedding (PCA PCoA t-SNE)

Studying the population structure of a meta-population can deepen our understanding of the stratification of the population and the migration of individuals in a population. In this study, we carried out principal component analysis (PCA), principal coordinates analysis (PCoA), and t-distributed stochastic neighbour embedding (t-SNE) analysis on all breeds. PCA was based on the eigenvector obtained from GCTA software (version 1.91.6) [19] and was analysed and plotted using the R program. Although PCoA [20] is similar to PCA, unlike PCA, it changes the coordinate system while the relationship between the sample points remains the same. t-SNE [21] is a machine learning algorithm for dimensionality reduction, and it is very suitable for dimensionality reduction of high-dimensional data to 2 or 3 dimensions for visualization. PCoA and t-SNE were performed by using the R packages “vegan” [22] and “Rtsne” [23], respectively.

### Partial least squares (PLS) method

We used the PLS method to explore the unique characteristics of each breed compared to other breeds. The principle of using the PLS method to detect unique characteristics is briefly described as follows:

First is the determination of the response variable y according to the breed category,

$$ y=\left\{\begin{array}{c}1,\mathrm{population}1\\ {}0,\mathrm{population}2\end{array}\right. $$

The response variable y is assigned a value of 1 when a breed has been determined as the study object. The remaining 23 breeds are assigned a value of 0. This was performed recursively for each breed to identify each breed’s unique SNPs compared to the other 23 breeds. The theory and feasibility of PLS applied to selection signature detection between populations can be found in Sun’s [8] paper.

### Functional annotation and enrichment analysis

We performed an additional analysis to further elucidate the biological functions of specific SNPs in this study. First, we found their corresponding genes using gene annotation data for pigs extracted from the Ensembl Genes database (http://asia.ensembl.org/info/data/index.html). Then, GO term and KEGG pathway analyses were performed using the KEGG Orthology-Based Annotation System [24] (KOBAS, http://kobas.cbi.pku.edu.cn/kobas3). By performing a further functional annotation of each breed’s specific and significant SNPs, we were able to identify some unique modules for each breed and the genetic variation associated with the breed’s important phenotypic trait.

### IPA analysis

Finally, to reveal the network of interactions and relationships between molecular products formed by the detected genes within a defined functional area, a gene interaction network diagram was constructed. IPA (Ingenuity Pathways Analysis, www.ingenuity.com) software was used to construct interaction network diagrams for the specific significant genes identified in each of the breeds. In the network diagram, genes, proteins, and chemicals are represented in different shapes. IPA uses a network generation algorithm to divide the network graph between molecules into multiple networks and score each network. The score is based on the hypergeometric distribution and the negative logarithm of the significance level obtained by Fisher’s exact test on the right tail. All the networks are ranked using score values.

## Supplementary Information

**Additional file 1.** Significant genes of 24 breeds.

**Additional file 2.** Significant GO Term and KEGG Pathway of 24 breeds.

**Additional file 3.** Significant ingenuity pathway analysis molecular networks of 24 breeds.

**Additional file 4.** The origin of all the pigs in our study.

## Data Availability

All SNP data supporting this study’s conclusions are available in the Open Science Framework (https://osf.io/wc9nk/?view_only=c5e79ade506b4effa622f09f1967e7e6) and a private website (https://jbox.sjtu.edu.cn/l/uoaCjx). Some data from previous studies are available under the Bioproject number PRJNA436152, PRJNA281578 and PRJNA471328.

## Abbreviations

GGRS: Genotyping by genome reducing and sequencing
SNP: Single nucleotide polymorphism
PCA: Principal component analysis
PCoA: Principal coordinates analysis
t-SNE: T-distributed stochastic neighbor embedding
PLS: Partial least squares
GO Term: Gene Ontology Term
KEGG: Kyoto Encyclopedia of Genes and Genomes
IPA: Ingenuity Pathways Analysis
