# Supplementary material for: Identifying the unique characteristics of the Chinese indigenous pig breeds in the Yangtze River Delta region for precise conservation
Source: BMC Genomics. 2021 Mar 2;22:151. doi: 10.1186/s12864-021-07476-7 (PMC7927379; doi:10.1186/s12864-021-07476-7)
Supplement: Supplementary file 4 — Additional file 4. The origin of all the pigs in our study. [file 12864_2021_7476_MOESM4_ESM.docx]

**Additional file 4:**

**The origin of all the pigs in our study**

| **Region** | **Breed** | **Code** | **Farm (Origin)** |
| --- | --- | --- | --- |
| Western | Duroc | D | Shanghai Xiangxin Livestock Co., Ltd. |
|  | Landrace | L | Shanghai Xiangxin Livestock Co., Ltd. |
|  | Yorkshire | Y | Shanghai Xiangxin Livestock Co., Ltd. |
|  | Pietrain | P | Shanghai Xinnong Group |
|  | Berkshire | B | Shanghai Wangu Pig Breeding Co., Ltd. |
| Jiangsu | Small Meishan | SMS | Taicang Pig Farm |
|  | Mi | MI | Jintan Mi Pig Original Breeding Farm |
|  | Erhualian | EH | Changshu Livestock and Poultry Seed Co., Ltd. |
|  | Dongchuan | DC | Taixing, Rugao |
|  | Huaibei | HB | Donghai Pig Farm |
|  | Hongdenglong | HD | Jiangsu |
|  | Jiangquhai | JQ | Jiangquhai Pig Farm |
|  | Shan | SZ | Shanzhu breeding center |
| Zhejiang | Bihu | BH | Lishui Bihu |
|  | Chunan | CA | Zhejiang Chunan |
|  | Chalu | CL | Ninghai, Chalu |
|  | Jinhualiangtouwu | JHL | Zhejiang Jiahua Pig Farm |
|  | Lanxi | LX | Lanxi, Yongchun |
|  | Shengxianhua | SH | Shengxian, Xinchang |
|  | Jiangxing Black | JX | Zhejiang Jiaxing Shuangqiao Farm |
| Shanghai | Middel Meishan | MMS | Meishan Pig Breeding Center, Jiading District, Shanghai |
|  | Shawutou | SW | Chongming County Breeding Farm, Shanghai |
|  | Fengjing | FJ | Shanghai Jinshan Breeding Center |
|  | Pudong White | PD | Shanghai Puhui Pudong White Pig Breeding Co., Ltd. |
